# Imaging the Anterior Segment: High-Frequency Ultrasound and Anterior Segment OCT

**DOI:** 10.1155/2013/398715

**Published:** 2013-12-18

**Authors:** Norma Allemann, D. Jackson Coleman, Charles J. Pavlin, David Huang

**Affiliations:** ^1^Department of Ophthalmology and Visual Sciences, FEDERAL University of São Paulo—UNIFESP, São Paulo, SP, Brazil; ^2^New York-Presbyterian Hospital at The Edward S. Harkness Eye Institute of Columbia University, New York, NY, USA; ^3^Department of Ophthalmology and Vision Sciences, University of Toronto, Toronto, ON, Canada; ^4^OHSU Oregon Health & Science University, Casey Eye Institute, Portland, OR, USA

This special issue was meant to demonstrate new areas of interest for the anterior segment imaging techniques as high-frequency ultrasound and anterior segment optical coherence tomography (OCT).

OCT is a rapidly developing technology that brought to the clinical practice high definition images of the anterior segment, including angle, providing corneal pachymetry maps and anterior chamber internal measurements, real-time images not needing a contact to the eye. This special issue brings novel applications for the OCT technology as aid in fitting prosthetic devices to treat corneal diseases, its utilization in pediatric ophthalmology, and papers evaluating glaucoma patients with AS-OCT and allowing the measurement of angle and the evaluation of the patency of a aqueous tube shunt.

In corneal procedures, OCT plays an important role as an easy and effective imaging technique, allowing the measurement of corneal scar thickness to plan for surgical procedures, providing the overall thickness mapping the areas of thinning or thickening and evaluating surgical procedures.

Authors suggested that limitations to the OCT technology include highly pigmented, thick, and deeply located lesions. The comparison of OCT and high-frequency ultrasound in anterior segment tumors pointed out that OCT is not able to evaluate the entire thickness of a lesion presented as solid melanocytic iris or ciliary body lesion and solid-cystic iris lesion (Bianciotto C et al., 2011), due to light attenuation, impeding the evaluation of anatomical relationship to adjacent structures and a correct measurement.

Considering superficial lesions as conjunctival nevi and squamous cell carcinomas, light attenuation can impede the evaluation of the whole thickness with anterior segment OCT and impede the evaluation of a scleral invasion (Shields C et al., 2011).

In the cases where AS-OCT cannot be utilized or does not provide adequate evaluation of the lesion and adjacent structures, ultrasound biomicroscopy (UBM) or high-frequency ultrasound (HFU) is indicated. There are cases in which both technologies do not allow evaluation, such as lesions containing high density of keratin or calcium.

*Norma Allemann* *Norma Allemann* *D. Jackson Coleman* *D. Jackson Coleman* *Charles J. Pavlin* *Charles J. Pavlin* *David Huang* *David Huang*

